# Multiple Dissociations in Patients With Disorders of Body Awareness: Implications for the Study of Consciousness

**DOI:** 10.3389/fpsyg.2018.02068

**Published:** 2018-10-26

**Authors:** Gabriella Bottini, Francesca Giulia Magnani, Gerardo Salvato, Martina Gandola

**Affiliations:** ^1^Department of Brain and Behavioral Sciences, University of Pavia, Pavia, Italy; ^2^NeuroMI—Milan Center for Neuroscience, University of Milano Bicocca, Milan, Italy; ^3^Cognitive Neuropsychology Centre, ASST Grande Ospedale Metropolitano Niguarda, Milan, Italy

**Keywords:** awareness, anosognosia for hemiplegia, somatoparaphrenia, caloric vestibular stimulation, consciousness

Elucidating the nature of consciousness has become one of the most relevant challenges in modern neuroscience. The study of patients with brain damage who exhibit selective impairments in awareness has contributed significantly to redefining the concept of consciousness, shedding light on at least two interesting aspects of its organization: the presence of behavioral and anatomical domain-specific dissociations and the possibility of modulating disturbances in awareness via both central and peripheral stimulations (e.g., caloric vestibular stimulation^1^—CVS—or transcranial Direct Current Stimulation^2^—tDCS). Evidence emerging from these lines of research has provided information regarding the nature of conscious processes, their neural substrates, and their associated physiological processes.

Anosognosia for hemiplegia (AHP), in which patients with brain damage deny the presence of their contralesional motor deficits (Babinski, 1914), represents how the conscious experience may be finely segmented. Indeed, motor anosognosia can manifest in a limb-specific and modality-specific fashion: For instance, it may affect the upper or lower limbs only (Von Hagen and Ives, 1937; Berti et al., 1996; Moro et al., 2011) and may concern either motor or sensory impairments (i.e., dissociations between AHP and anosognosia for hemianesthesia: AHA; see for example Marcel et al., 2004; Spinazzola et al., 2008; Pia et al., 2014). Moreover, previous studies have reported double dissociations between anosognosia and neglect (Bisiach et al., 1986; Berti et al., 1996; Marcel et al., 2004), revealing that AHP does not depend on the attentional deficits typical of Unilateral Spatial Neglect (Bisiach et al., 1986; Berti et al., 1996; Marcel et al., 2004). The existence of these dissociations suggests that brain lesions selectivity affect conscious processes, rather than inducing general and global impairments in awareness. Thus, patients may be aware of one deficit (e.g., hemianesthesia) yet unaware of another (e.g., hemiplegia).

The use of structured interviews (see for example Bisiach et al., 1986; Berti et al., 1996) and systematic experimental paradigms (Jenkinson and Fotopoulou, 2010) has revealed that motor denial can manifest in various ways. For example, when required to perform a specific action (such as clapping), some patients became aware that they are unable to execute the movement (moderate AHP, score 2/2, in Bisiach et al., 1986; emergent awareness in Moro et al., 2011), while others claim to have performed the action despite their paralysis (severe AHP, score 3/3 in Bisiach et al., 1986). These differences in awareness have been classically conceptualized as different degrees of severity (Bisiach et al., 1986). Alternatively, these two behaviors may be related to different cognitive mechanisms. Among patients who claim they have performed the movement despite demonstrations that no movements have occurred (i.e., those with apparently more severe anosognosia), this obstinate motor delusion seems to be related to the loss of on-going monitoring mechanisms. In other words, this denial is embedded into the neural systems subserving the comparison between the intended and performed movements (Frith et al., 2000; Blakemore et al., 2002). Conversely, among patients who realize they have not moved after failure of the action, the initial denial of impairment may depend on the semantic knowledge that the body segments can move, and on the memory of being able to move prior to the occurrence of the brain lesion (Marcel et al., 2004). Thus, the false belief of preserved motor competence is supported by a left hemisphere “*narrator”* telling what he knows about bodily functions (Geschwind, 1965).

Previous research has also revealed interesting dissociations between *implicit* and *explicit* awareness: Indeed, patients with AHP may have implicit knowledge of their deficit, which is otherwise denied explicitly (see for example Marcel et al., 2004; Fotopoulou et al., 2010; Vocat and Vuilleumier, 2010; Moro et al., 2011). Explicit awareness can be explored directly by asking patients to verbally declare whether they can move, while implicit awareness is generally indirectly inferred based on behavioral observations (Fotopoulou et al., 2010; Moro et al., 2011). Several reports have indicated that different levels of awareness may coexist within the same patient: “Patients who verbally deny their hemiplegia usually do not object of being confined to bed” (Bisiach and Geminiani, 1991). More recently, this dissociation has been explored systematically using either verbal (Fotopoulou et al., 2010) or motor paradigms (Moro et al., 2011). These studies also provide partially convergent anatomical patterns of the dissociation between explicit and implicit awareness.

Anatomical evidence also supports the modular organization of consciousness. In 2005, Berti et al. (2005) demonstrated that AHP occurs due to lesions of the cerebral regions that monitor motor functions, such as the premotor cortex, rather than damage to a general supramodal neural system. Notably, the brain lesion of a single patient, who exhibited anosognosia without signs of neglect, overlapped with the damage associated with AHP in the between groups comparisons, supporting the hypothesis that motor-monitoring deficits can be disentangled from USN. However, Karnath et al. (2005) reported different results, instead emphasizing the role of the posterior insula in AHP. Further evidence supports the notion that different forms of anosognosia correspond to discrete anatomical substrates depending on the sensory-motor disorders observed. These studies have suggested that AHA is associated with lesions of brain areas such as the putamen, which is well known to subserve sensory processing, contrasting somewhat with the regions involved in AHP (Spinazzola et al., 2008; Pia et al., 2014).

However, more recent studies (Fotopoulou et al., 2010; Vocat et al., 2010; Moro et al., 2011, 2016) have provided a more complex picture of the brain regions involved in awareness, suggesting that AHP is a multi-component disorder caused by lesions of complex and distributed cortical-subcortical anatomical networks, rather than isolated regions (see discussion in Fotopoulou, 2014). Given the available evidence, combined use of the lesion-symptom method and modern neuroanatomical approaches such as fMRI/resting-state fMRI, diffusion tensor imaging, and fiber tractography may allow researchers to overcome limitations associated with defining large-scale networks involved in complex cognitive functions (e.g., body and motor awareness), based on classical clinical anatomical correlations alone (Catani et al., 2012; Thiebaut de Schotten and Foulon, 2018). Elucidating these networks may advance our understanding of the neural and physiological bases of consciousness (see for example Gandola et al., 2014a).

Disorders of consciousness may affect different levels of body representation. AHP is frequently associated with asomatognosia (see Jenkinson et al., 2018) and somatoparaphrenia (SP), which is characterized by the delusion that the paralyzed limb does to not belong to oneself (Gerstmann, 1942). In AHP and SP, impairments in consciousness are associated with bodily functions and body ownership, respectively. Rare cases of SP without AHP have been described (see for example Invernizzi et al., 2012; Moro et al., 2016). Invernizzi et al. (2012) demonstrated that SP is mainly associated with lesions to the right thalamus, basal ganglia, and posterior limb of the internal capsule, sparing the dorsal and premotor regions typically associated with AHP (Berti et al., 2005). The lesional pattern defines the neuropsychological manifestations observed, although the level of interdependence among the different components contributing to mental representation of the body remains unknown. The existence of patients who, surprisingly enough, deny ownership of their paralyzed limb although they recognize that they cannot move it may be explained by the influence of selective awareness modules on unshared senses of agency and body ownership as independent components, supporting an independent rather than additive model of body representation (Tsakiris et al., 2006; Invernizzi et al., 2012). More recently, Moro et al. (2016) revealed that disturbed sensation of limb ownership (DSO) is associated with more medial and subcortical lesions (mainly involving the basal ganglia and surrounding white matter) than those involved in AHP. This evidence supports the existence of a neural dissociation between DSO and AHP in contrast to previous evidence, which suggested a crucial role of the right posterior insula for both the sense of limb ownership and motor anosognosia (Baier and Karnath, 2008).

Extensive research has suggested that central and peripheral stimulation (e.g., CVS, tDCS) can induce transient and selective remission in patients with various disorders of awareness (e.g., USN, anosognosia, and somatoparaphrenia), supporting the notion that conscious experience can be selectively modified. For instance, CVS may restore motor awareness (see for example Vallar et al., 1990, 2003; Bisiach et al., 1991; Rode et al., 1992), the sense of body ownership (Bisiach and Geminiani, 1991; Rode et al., 1992; Salvato et al., 2016), and sensory perception (Vallar et al., 1990, 1993; Bottini et al., 1995, 2005). Moreover, the effects of CVS on these disorders suggest that physiological components play a role in the conscious bodily experience, interacting with cognitive processes. In our recent study, for example, we utilized CVS in a patient with SP, who regained the sense of body part ownership following stimulation. We also observed an increase in body temperature following CVS, which correlated with the temporarily restored sense of limb ownership (Salvato et al., 2018). We speculated that this effect may have been due to partial overlap between the neural correlates of body ownership, thermoregulatory control, and the area stimulated by left-CVS. Alternatively, CVS may modulate interoceptive signals (e.g., body temperature), which may in turn have increased the sense of body ownership. Interestingly, in healthy volunteers, CVS induces a temperature drop in both arms, which is accompanied by bilateral improvements in tactile acuity (Sedda et al., 2016). Other research has indicated that selective remission can be induced in patients with disorders of awareness using simple verbal (Case F. B. in Bottini et al., 2002) or spatial manipulations (Salvato et al., 2016) or using mirror techniques (Jenkinson et al., 2013), highlighting the malleability of conscious processes.

Moreover, tDCS is effective in modulating disorders of motor awareness. For instance, tDCS over the right premotor cortex has been shown to induce selective remission of AHP (Gandola et al., 2014b). Such improvements manifested only when the patient was requested to perform the action (online judgment) with his eyes open, while motor anosognosia remained when the patient judged non-attempted actions (offline condition) and during the eyes-closed online condition. Selective modulation of motor awareness has also been observed in healthy volunteers, revealing that posterior parietal modulation interferes with non-intentional movement awareness, while premotor cortex modulation interferes with intentional movement awareness (Bolognini et al., 2016; Bruno et al., 2017).

In conclusion, although the neurophysiological organization of awareness into discrete neural systems may explain the multiple dissociations of symptoms observed in patients with brain damage, alternative hypotheses have also been proposed. Recently, the influential theoretical framework, which is based on the free-energy principle and Bayesian inference (Friston, 2005), has been applied to the study of AHP (Fotopoulou, 2014, 2015) and self-recognition (Apps and Tsakiris, 2014). This framework may provide an alternative to highly modular models of motor and self-awareness and may represent a unified explanation of the clinical variability/dissociations of such deficits (Fotopoulou, 2014, 2015).

## Author contributions

GB, FGM, GS, and MG equally contributed in drafting and revising the final version of the manuscript.

### Conflict of interest statement

The authors declare that the research was conducted in the absence of any commercial or financial relationships that could be construed as a potential conflict of interest.

## Footnotes

^1^CVS involves irrigation of the left or right external auricular canal with warm or cold water. This produces effects on several cognitive processes by eliciting neural responses from vestibular neurons, which are strongly interconnected with higher-order brain regions (see review in Bottini and Gandola, 2015).

^2^By applying a low-intensity electric current to the scalp, tDCS allows for controlled interference with brain activity. Inhibitory (cathodal) or excitatory (anodal) modulation can be induced in the stimulated brain region (Fregni et al., 2015).
